# A high throughput screen for pharmacological inhibitors of the carbohydrate response element

**DOI:** 10.1038/s41597-023-02596-z

**Published:** 2023-10-04

**Authors:** Shaochen You, Michael J. Bollong

**Affiliations:** Department of Chemistry, The Scripps Research Institute, La Jolla, California 92037 USA

**Keywords:** Chemical genetics, Chemical tools

## Abstract

A central regulator of metabolism, transcription factor carbohydrate response element binding protein (ChREBP) senses and responds to dietary glucose levels by stimulating the transcription of glycolytic and lipogenic enzymes. Genetic depletion of ChREBP rescues β-cell dysfunction arising from high glucose levels, suggesting that inhibiting ChREBP might represent an attractive therapeutic approach to manage diabetes and other metabolic diseases. However, the molecular mechanisms governing ChREBP activation are poorly understood and chemical tools to probe the cellular activity of ChREBP are lacking. Here, we report a high-throughput pharmacological screen in INS-1E β-cells that identified novel inhibitors of ChREBP-driven transcription at carbohydrate response element sites, including three putative covalent inhibitors and two likely non-covalent chemical scaffolds. This work affords a pharmacological toolkit to help uncover the signaling logic controlling ChREBP activation and may ultimately reveal potential therapeutic approaches for treating metabolic disease.

## Background & Summary

**Fig. 1:**
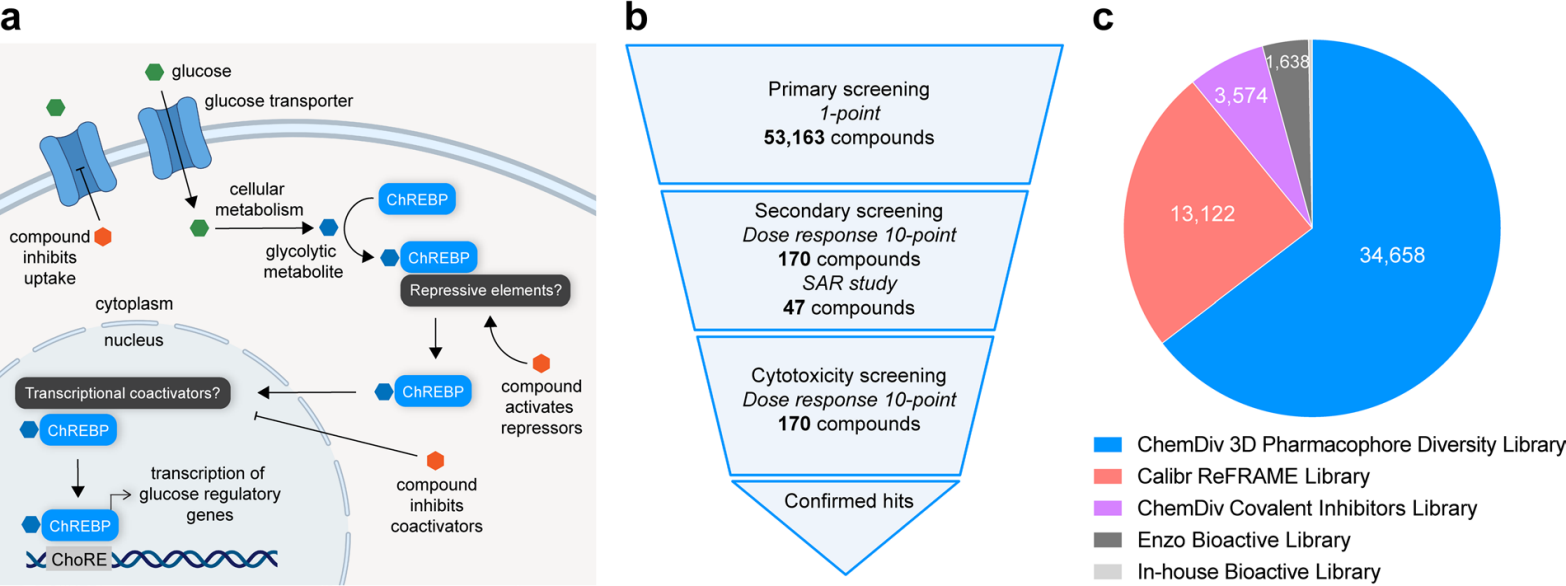
Overview of the study. (**a**) Schematic depicting the current state of understanding how ChREBP is activated in the cell. Unknown glycolytic metabolites are sensed by ChREBP, which subsequently translocates to the nucleus to bind to cognate ChoRE sequences in transcriptionally responsive loci. Small molecule inhibitors might act by inhibiting glucose uptake into the cell, by activating repressive elements, or by inhibiting transcriptional coactivators necessary for ChREBP activity. (**b**) Schematic of the workflow for the ChoRE-LUC reporter-based high-throughput screen. INS-1E ChoRE-LUC cells were screened against various libraries. Hits were identified and subject to secondary dose-response screening and cytotoxicity screening. (**c**) Pie chart depicting the number of compounds contained within each of the primary screen libraries evaluated in this work.

Glucose serves as a ubiquitous source of energy for all organisms and its regulation is of central importance to organismal function. Indeed, prolonged elevation of blood glucose levels dramatically increases risk of metabolic diseases such as type 2 diabetes, obesity, and nonalcoholic fatty liver disease. Accordingly, pharmacological modulation of glucose responsive signaling pathways may provide novel therapeutic strategies for treating these disorders. One such metabolic regulator that responds to changing glucose levels is the transcription factor carbohydrate response element binding protein (ChREBP). ChREBP is a member of the Mondo family of basic helix-loop-helix leucine zipper (bHLH-zip) transcription factors^1^. It contains two nuclear export signals and a nuclear localization signal in the N-terminus region and the bHLH-zip domain as well as a Zip-like domain in its C-terminus, which interacts with transcriptional co-activators like Max like protein x (Mlx)^2^ and p300/CBP^3^. ChREBP regulates multiple signaling and metabolic pathways in several tissues including the liver, white and brown adipose tissue, small intestine, and pancreatic islets^4^. During fasting conditions, ChREBP is inactivated via phosphorylation by protein kinase A^5^ and AMP activated protein kinase^6^, leading to its cytosolic retention. In response to carbohydrates however, certain glucose derived metabolites bind to the glucose-response activation conserved element (GRACE) on the N-terminus of ChREBP^7^. This, in conjunction with dephosphorylation by protein phosphatase 2A^8^, activates ChREBP, promoting its dimerization with Mlx^9^ and translocation to the nucleus, where it undergoes *O*-GlcNacylation^10^ and acetylation^11^ to promote binding to its cognate carbohydrate response element (ChoRE) in the loci of target genes (Fig. 1a). Previous studies have proposed glucose-6-phosphate^12^ or xylulose-5-phosphate^13^ as a potential glucose derived metabolites with ChREBP activating potential; however, the specific metabolite that ChREBP directly senses remains unclear.

ChREBP is primarily expressed in organs with high levels of *de novo* lipogenesis. In the liver, ChREBP augments lipogenesis in hyperglycemic states by upregulating transcription of key enzymes, including pyruvate kinase, fatty acid synthase, acetyl-CoA carboxylase^14^, and indirectly through ATP-citrate lyase (through its upregulation of the branched-chain ketoacid dehydrogenase complex)^15^. Secretion of very low density lipoprotein is also controlled in part by ChREBP^16^ as hepatic ChREBP knockdown decreases microsomal triglyceride transfer protein levels in the liver^17^. ChREBP overexpression in the liver of mice has been shown to increase hepatic steatosis^18^ and genetic ChREBP ablation in the liver reverses hepatic steatosis^19^. In addition, ChREBP modulates fructose tolerance in the liver by increasing expression of fructokinase and triose kinase^20^. In the small intestine, ChREBP drives expression of glucose transporters 2 and 5 as well as ketohexokinase and sucrose-isomaltase, promoting sugar absorption^21^.

ChREBP is also critically important in regulating pancreatic function. In pancreatic β-cells, ChREBP drives expression of thioredoxin-interacting protein (TXNIP), the accumulation of which causes β-cell damage^22^ by increasing inflammation through activation of the NLRP3 inflammasome^23^, promoting apoptosis via inhibition of thioredoxin^24^, and decreasing insulin sensitivity by promoting the internalization of glucose transporters^25^. Therefore, inhibiting expression of TXNIP may protect β-cells from the cytotoxic effect of high glucose levels, enabling β-cell expansion and increasing insulin production. Notably, knockdown of ChREBP and/or TXNIP has been shown to improve long-term glucose tolerance and insulin sensitivity in mouse models of obesity, suggesting that decreasing TXNIP levels pharmacologically could be a potential therapeutic route for treating diabetes and its complications. To date, W2476 is the only synthetic small molecule that has been reported to inhibit TXNIP expression^26^. Mechanistically, W2476 was found to inhibit the phosphorylation of forkhead box O1 transcription factor (FOXO1), which increases FOXO1 binding to the TXNIP promoter, therefore preventing ChREBP binding^27^. Given this compound inhibits FOXO1 signaling globally, W2476 does not necessarily act as a specific inhibitor of ChREBP activity, and thus the discovery of additional pharmacological agents along with their associated ligandable targets will be essential in helping further define the logic of ChREBP activation.

**Fig. 2:**
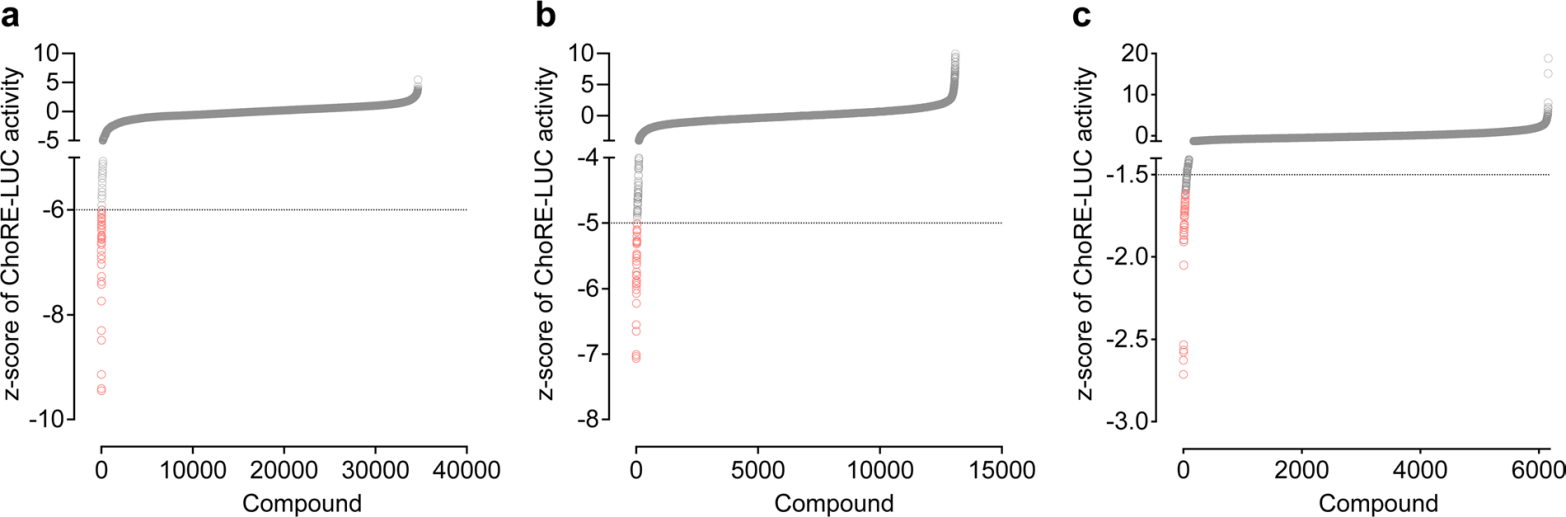
Results of primary screening. Ranked plot of evaluated compounds sorted by z-scores of ChoRE-LUC activity from primary screening of the ChemDiv 3D Pharmacophore Diversity Library (**a**; z-score cutoff = −6), the ReFRAME Library (**b**; z-score cutoff = −5), the ChemDiv Covalent Inhibitor, Enzo, and In-house bioactive libraries (**c**; z-score cutoff = −1.5).

Here, we report results from a cell-based high-throughput screen of 53,163 compounds aimed at identifying small molecule inhibitors of ChoRE driven transcription (Fig. 1b,c). We generated an INS-1E cell line stably expressing a ChoRE-dependent luciferase reporter (ChoRE-LUC) and developed a 384-well compatible screening assay to identify compounds that inhibited transcriptional activity at ChoREs. The libraries that were screened were the comprehensive drug repurposing library ReFRAME, a structurally diverse pharmacophore library, a covalent inhibitors library, and known bioactive small molecule libraries: the Enzo library and an In-house bioactive library (Fig. 2). Following primary screening, top hits from each library were re-tested in dose response for modulating ChoRE-LUC activity and cytotoxicity. Further structure-activity relationship (SAR) analysis using commercially available analogs was performed on hits from the covalent inhibitor library.

## Methods

### Compound libraries

High-throughput screening was performed using five compound libraries, screened at the highest concentration available from the source compound plates. The Calibr ReFRAME library contains 13,122 compounds and was screened at 2 µM final concentration. The Calibr ReFRAME library was supplied by Calibr. Its construction and composition have been described in the literature^28^. The 3D-Pharmacophore Based Diversity Library contains 34,658 compounds screened at 20 µM final concentration and was obtained from the commercial vendor ChemDiv. The Covalent Inhibitors Focused Library contains 3,574 compounds screened at 2 µM final concentration and was also obtained from ChemDiv. The Enzo library contains 1,638 compounds and was obtained from Enzo Life Sciences. The In-house bioactive library contains 171 compounds and was obtained from several chemical suppliers including Tocris and Sigma. The compounds were stored in DMSO at −20 °C.

### Cell culture

Rat INS-1E cells were a gift from Weijun Shen (Calibr, a division of Scripps Research). INS-1E cells were maintained in RPMI medium (Corning, 10-040-CV) supplemented with 10% fetal bovine serum (FBS, Gibco, 10438-026), 10 mM HEPES (Corning, 25-060CI), 1 mM sodium pyruvate, (Gibco, 11360070), and 1% penicillin/streptomycin (Gibco, 15070063). HEK293T cells were obtained from the American Type Culture Collection (ATCC, CRL-11268) and were maintained in DMEM medium (Corning, 10-013-CV) supplemented with 10% FBS and 1% penicillin/streptomycin.

### Reporter plasmids

pGL4-ChoRE-LUC reporter plasmids were generated from codon-optimized ChoRE promoter constructs (obtained from Integrated DNA Technologies as gBlock HiFi Gene Fragments) cloned into the pGL4 backbone from pGL4.40 (Promega, E4131) using the NEBuilder HiFi DNA Assembly Kit (NEB, E5520S). pGF1-ChoRE WT-LUC plasmid was generated in the same way using the pGreenFire backbone (System Biosciences). For transient transfection of reporter plasmids in 384-well plate format, INS-1E WT cells (5000 cells in 40 µL assay medium per well) were transfected with 100 ng plasmid DNA in 10 µL OptiMEM media (Gibco, 31985062) containing a 1:3 ratio (µg DNA:µL reagent) of FuGENE HD (Promega, E2311) at the time of cell seeding.

### Stable cell lines

For production of lentivirus, 1 × 10^6^ HEK293T cells were plated on poly-D-lysine coated 10 cm plates in growth medium and transfected with 2 µg of pGF1-ChoRE WT-LUC and 2 µg of each packaging plasmid (pMD2.G and psPAX2, Addgene #12259, #12260) with 24 µL of FuGENE HD transfection reagent in 600 µL of OptiMEM medium at the time of cell seeding. 10 mL of cell culture supernatant was harvested after 48 h and clarified via centrifugation (3 min, 300 x*g*) and filtration with a 0.45 µm syringe filter, then concentrated to 1 mL using a 30 kDa MWCO centrifugal filter (Amicon). The concentrated supernatant was added to 4 × 10^5^ INS-1E cells one day after seeding in a six-well plate. After 72 h, INS-1E ChoRE-LUC stable cell lines were selecting using puromycin (1.6 µg/mL) for 48 h. Fluorescent activated cell sorting (FACS) was used isolate single clones expressing green fluorescent protein.

### High-throughput screening

For ReFRAME library screening, 10 nL of 2 mM DMSO stock concentrations of compound were prespotted into 5 µL of assay medium (RPMI with 2% FBS, 10 mM HEPES, 1 mM sodium pyruvate, 25 mM D-glucose (Sigma), and 1% penicillin/streptomcycin) in white 384-well plates (Grenier) using an Echo Acoustic Liquid Handler instrument (Labcyte) before the addition of INS-1E ChoRE-LUC cells (5000 cells in 45 µL assay medium per well). Cells were incubated for 24 h before 30 µL of BrightGlo Luciferase Assay solution (diluted 1:3 in water, Promega) was added to each well and luminescence signal values were recorded with an Envision plate reader (PerkinElmer). This protocol was used as the ReFRAME library was supplied from off-site and necessitated pre-spotting for efficient transfer of materials. For the screening of all other libraries, which was performed in-house, INS-1E ChoRE-LUC cells were plated in a white 384-well plate (5000 cells in 50 µL assay medium per well) and incubated for 24 h. 100 nL of DMSO stock concentrations of compound were transferred to each well using a Bravo instrument with a pintool head (Agilent). Cells were incubated for 24 h before 30 µL of BrightGlo Luciferase Assay solution was added to each well and luminescence signal values were recorded with an Envision plate reader.

### Cytotoxicity screening

INS-1E ChoRE-LUC cells were plated in a white 384-well plate (5000 cells in 50 µL assay medium per well) and incubated for 24 h. 100 nL of DMSO stock concentrations of compound were transferred to each well using a Bravo instrument (Agilent) affixed with a pintool head (V&P Scientific). Cells were incubated for 24 h before 30 µL of CellTiterGlo Cell Viability Assay solution (diluted 1:6 in water, Promega) was added to each well and luminescence signal values were recorded with an Envision plate reader.

### Data analysis

Reporter activity and cell viability data was normalized to the DMSO control wells in each plate. Ten or twelve dose-response points and three biological replicates were used to determine dose-response curves using the four-parameter inhibitor dose-response curve fitting function in Prism software (Graphpad). All graphed data are plotted as the mean and the s.e.m denoted with error bars.

### Chemicals used

W2476 was obtained from ChemDiv. CYC065, RO-4584820, and PCA 4248 were obtained from Cayman Chemical. All chemicals were dissolved in DMSO and used without further purification.

## Data Records

### Primary and secondary screening compounds

The compounds used in the primary and secondary luciferase reporter and viability assay screens are detailed in “Primary and Secondary Screening Compounds.xlsx” within figshare^29^. We provide compound ID, common identifiers, and chemical structures for the compounds used in the ReFRAME, ChemDiv 3D Pharmacophore, ChemDiv Covalent Inhibitors, Enzo, and In-house Bioactive libraries used in the primary and secondary screens. The table headings present in the data file are defined in Table 1.

**Table 1:** Table headings used for the “Primary and Secondary Screening Compounds.xlsx” file deposited on figshare^29^.

Variable	Unit	Explanation
Compound ID	Dimensionless	Unique arbitrary assigned identifier of the compound.
Plate	Dimensionless	Unique plate identifier.
Well	Dimensionless	Unique well identifier. Consists of a letter from A to P, followed by a number from 01 to 24.
PubChem CID	Dimensionless	PubChem Compound ID number (if applicable).
CAS Registry Number	Dimensionless	Chemical Abstracts Service Registry Number (if applicable).
ChEMBL ID	Dimensionless	ChEMBL database ID number (if applicable).
SMILES	Dimensionless	Canonical SMILE of the compound.
InChIKey	Dimensionless	International Chemical Identifier Key.
Name	Dimensionless	Common compound name (if applicable).
Hit	Dimensionless, yes or no entry	Describes if the compound is a hit by initial z-score cutoff.

### Primary screening ChoRE-LUC activity data

The primary luciferase reporter screen data set can be found in “Primary Screening ChoRE-LUC Activity Data.xlsx” within figshare^29^. We provide screen-wide normalized data, raw data, and median and mean z-scores for the ReFRAME, ChemDiv 3D Pharmacophore, ChemDiv Covalent Inhibitors, Enzo, and In-house bioactive libraries used in the primary screen. The table headings present in the data file are defined in Table 2.

**Table 2:** Table headings used for the “Primary Screening ChoRE-LUC Activity Data.xlsx” file deposited on figshare^29^.

Variable	Unit	Explanation
Compound ID	Dimensionless	Unique arbitrary assigned identifier of the compound.
Median z-score	Real number	Median z-score.
Mean z-score	Real number	Mean z-score.
Relative ChoRE-LUC Signal	Real number	Relative luminance value readout from the ChoRE-LUC reporter assay compared to DMSO at the assay screening compound concentration.
Absolute ChoRE-LUC Signal	Real number	Raw luminance value readout from the ChoRE-LUC reporter assay or cytotoxicity assay.

### Secondary dose response screening ChoRE-LUC activity data

The secondary dose response luciferase reporter screen data set can be found in “Secondary Dose Response Screening ChoRE-LUC Activity Data.xlsx” within figshare^29^. We provide dose-response normalized data and raw data for hits from the ReFRAME, ChemDiv 3D Pharmacophore, ChemDiv Covalent Inhibitors, ChemDiv Covalent Inhibitors SAR, and In-house Bioactive libraries. The table headings present in the data file are defined in Table 3.

**Table 3:** Table headings used for the “Secondary Dose Response Screening ChoRE-LUC Activity Data.xlsx” and “Secondary Dose Response Screening Viability Data.xlsx” files deposited on figshare^29^.

Variable	Unit	Explanation
Compound ID	Dimensionless	Unique arbitrary assigned identifier of the compound.
Replicate	Integer	Biological replicate number (if applicable).
*X* µM	Real number	Relative (compared to DMSO at compound concentration *X* µM) or raw luminance value readout from the ChoRE-LUC reporter assay or cytotoxicity assay.

### Secondary dose response screening viability data

The secondary dose response cytotoxicity screen data set can be found in “Secondary Dose Response Screening Viability Data.xlsx” within figshare^29^. We provide dose-response normalized data and raw data for hits from the ReFRAME, ChemDiv 3D Pharmacophore, ChemDiv Covalent Inhibitors, ChemDiv Covalent Inhibitors SAR, and In-house Bioactive libraries. The table headings present in the data file are defined in Table 3.

## Technical Validation

### Reporter optimization

**Fig. 3:**
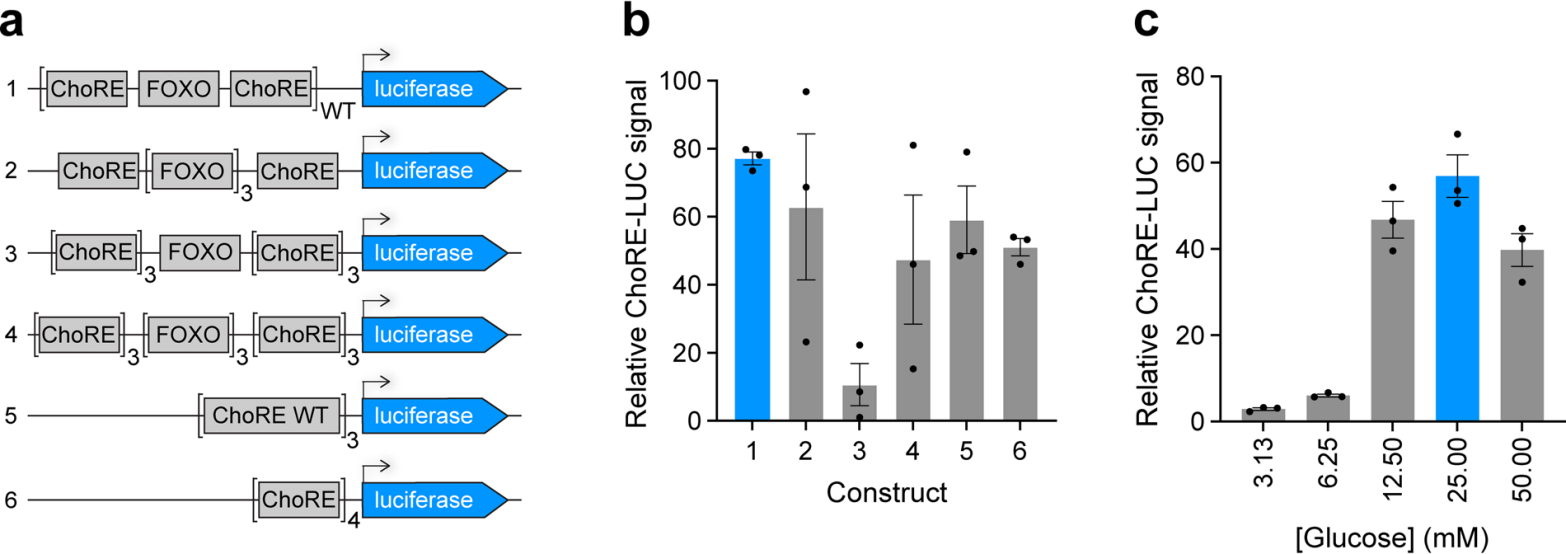
Optimization of the cellular ChoRE activity reporter. (**a**) Schematic of the six ChoRE-LUC reporter constructs tested. ChoRE WT refers to the ChoRE-FOXO-ChoRE sequence in construct 1. (**b**) Relative luminance values for the six ChoRE-LUC reporter constructs in INS-1E WT cells after 24 h in 25 mM glucose assay media. (**c**) Relative luminance values for the ChoRE WT reporter construct (construct 1) after 24 h in the indicated concentrations of glucose in the assay media.

To establish a reliable assay with a suitable dynamic range for high-throughput screening, we first optimized the composition of the DNA regulatory sequences within the ChoRE-LUC reporter. Previous studies showed that the minimal promoter required for ChoRE-driven TXNIP expression induction by glucose consists of a ChoRE sequence (two E-boxes, CACGTG, separated by 5 nucleotides), a CCAAT box, a FOXO1 binding site, an inverted CCAAT box, and a second degenerate ChoRE sequence^30^. To determine whether this combination of regulatory elements was robust enough to test general ChoRE activity, we constructed six pGL4-based luciferase reporter plasmids with different numbers and/or combinations of ChoRE and FOXO sequences from the promoter of the human TXNIP gene (Fig. 3a). The wild-type (WT) sequence represents the intact minimal promoter of the TXNIP gene (GRCh38.p14, chr1:145,996,639–145,996,759). The six plasmids were transiently transfected into INS-1E cells using cationic lipid-based transfection reagents and the induction of luciferase after 24 h in 25 mM glucose containing media was compared (Fig. 3b). In addition, we tested various glucose concentrations in the assay media to determine the concentration that results in the highest stimulation. We determined that the WT ChoRE-LUC construct in 25 mM glucose media resulted in the most maximal and consistent increase in luminance signal (Fig. 3c). The WT ChoRE-LUC construct was then used to generate an INS-1E stable cell line (INS-1E ChoRE-LUC) for screening in 384-well plate format. For high-throughput screening, the treatment time, serum percentage in media, and cell count per well were optimized further. The final assay design consisted of 5,000 INS-1E ChoRE-LUC cells per well of a 384-well plate in 25 mM glucose media supplemented with 2% FBS and a compound treatment time of 24 h.

### Screening assay

**Fig. 4:**
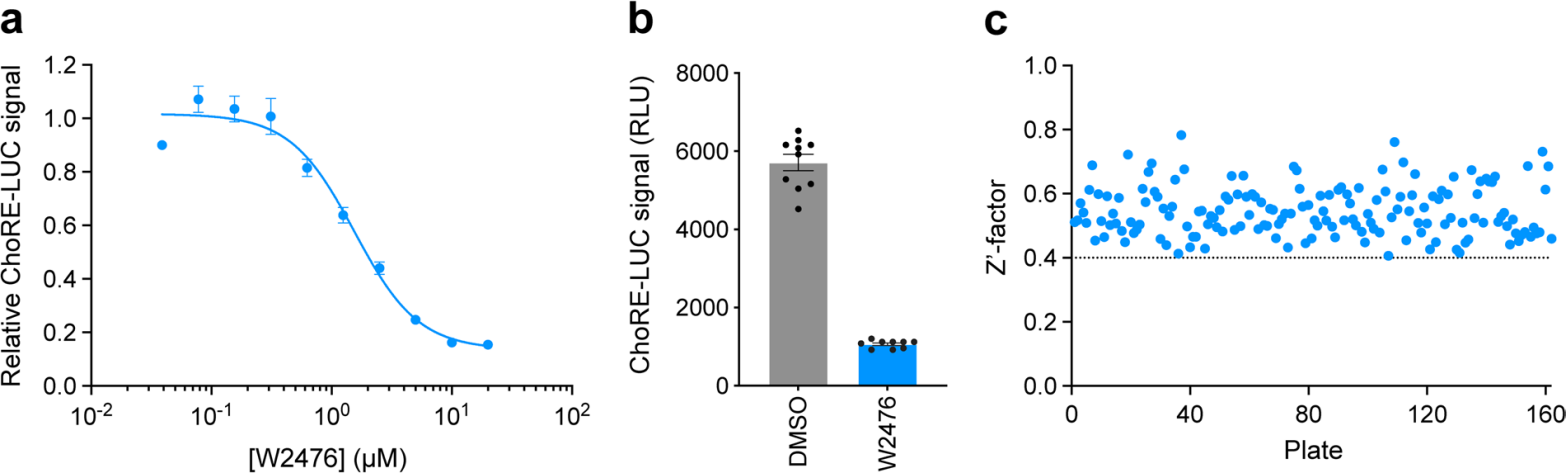
Assay validation. (**a**) Relative luminance values for INS-1E ChoRE-LUC cells treated for 24 h with W2476. (**b**) Absolute luminance values (RLU) for INS-1E ChoRE-LUC cells treated for 24 h with either DMSO or 10 µM W2476. Z’-factor = 0.51. (**c**) Plot of Z’-factors for each plate tested in the primary screen. Z’-factor cutoff = 0.4.

**Fig. 5:**
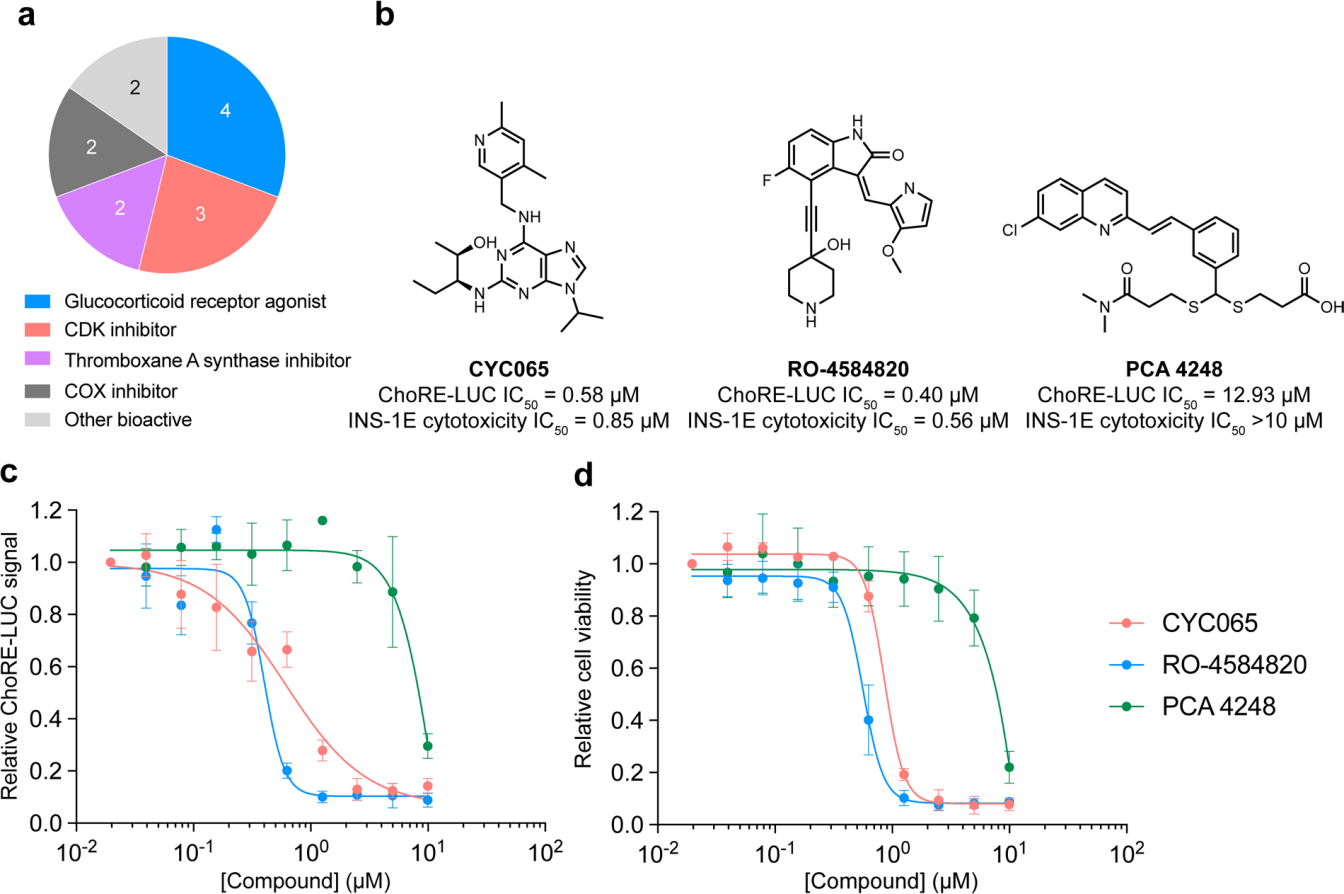
Results of the Calibr ReFRAME screen. (**a**) Annotated biological activity of hits from the primary ReFRAME screen. (**b**) Structures and cellular activities of the 3 top hits from the ReFRAME screen. (**c**) Relative luminance values for INS-1E ChoRE-LUC cells treated for 24 h with the indicated compounds. (**d**) Relative cell viability for INS-1E ChoRE-LUC cells treated for 24 h with the indicated compounds.

**Fig. 6:**
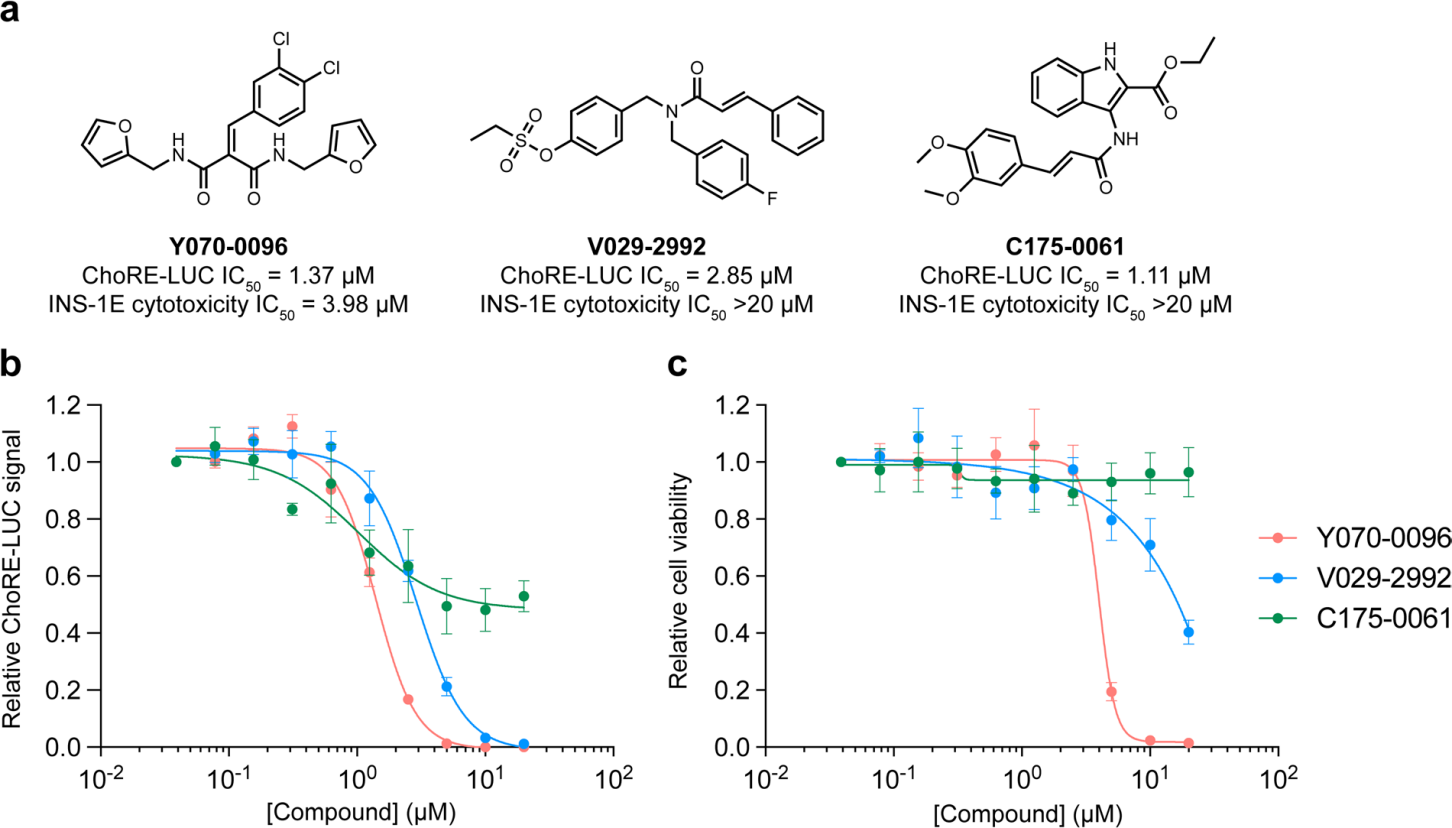
Results of the ChemDiv Covalent Inhibitor Library screen. (**a**) Structures and cellular activities of the 3 top hits from the covalent inhibitor library screen. (**b**) Relative luminance values for INS-1E ChoRE-LUC cells treated for 24 h with the indicated compounds. (**c**) Relative cell viability for INS-1E ChoRE-LUC cells treated for 24 h with the indicated compounds.

**Fig. 7:**
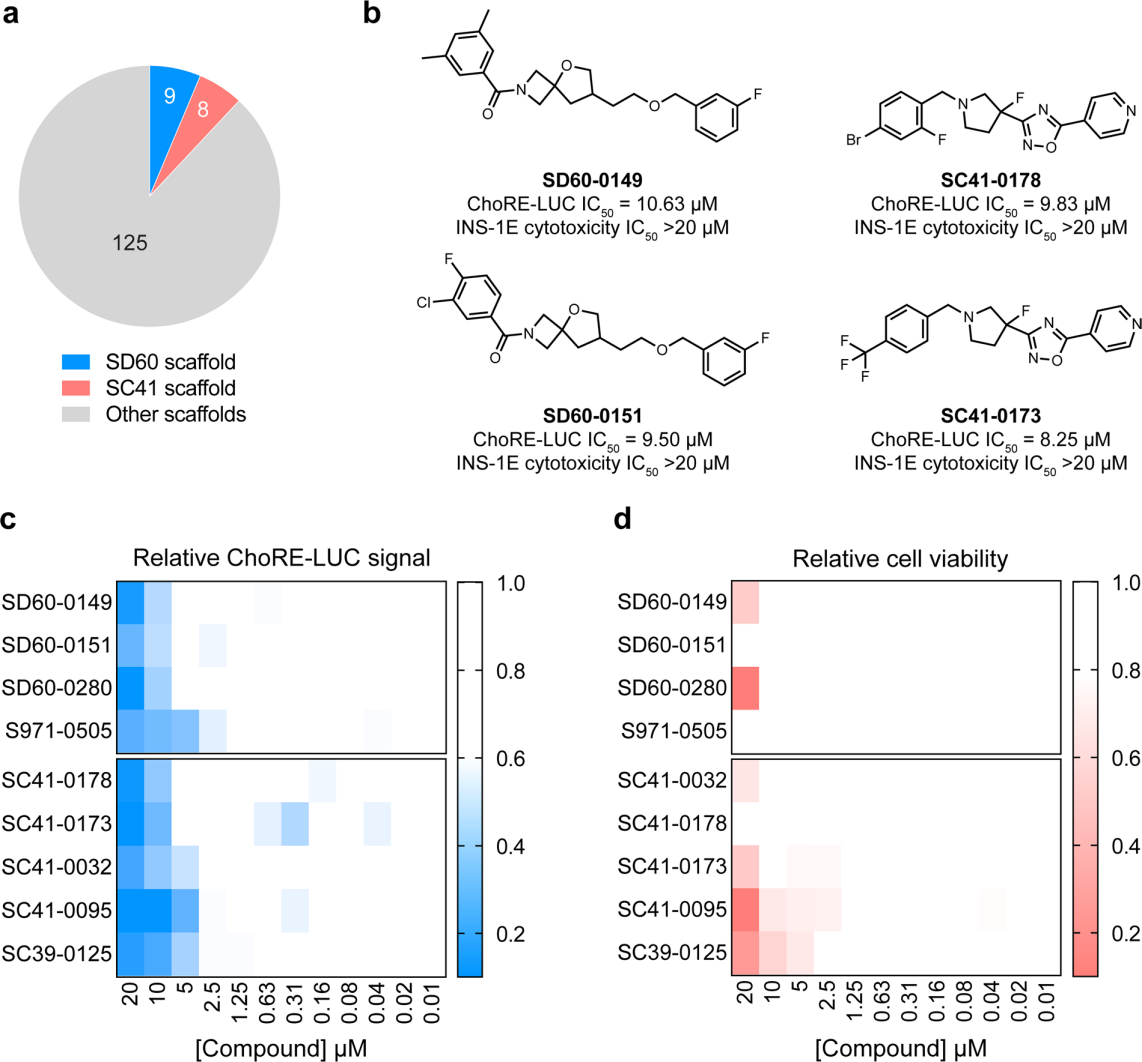
Results of the ChemDiv 3D Pharmacophore Diversity Library screen. (**a**) Distribution of hit scaffolds from the primary covalent inhibitor library screen. (**b**) Representative structures and cellular activities from the SD60 and SC41 scaffolds. (**c**) Relative luminance values for INS-1E ChoRE-LUC cells treated for 24 h with the indicated compounds. (**d**) Relative cell viability for INS-1E ChoRE-LUC cells treated for 24 h with the indicated compounds.

Pharmacological validation of the assay was performed using the known TXNIP expression inhibitor W2476. The ChoRE-LUC IC_50_ value (1.33 µM) obtained for W2476 in our assay was similar to the previously reported measurement (1.70 µM)^26^, indicating the assay was likely suitable for interrogating inhibitory effects on TXNIP expression via ChoREs (Fig. 4a). The primary screen was validated by calculating the Z’-factor for each plate (Fig. 4b). All 162 plates had a Z’-factor greater than 0.40, indicating good assay quality (Fig. 4c). The average Z’-factor for all plates was 0.55. The Z’-factor per library is shown in Table 4. Hit compounds from each library were identified by z-score cutoff values (Table 4). The primary screen returned 170 hits (0.31% hit rate) which were re-purchased from chemical suppliers and subjected to secondary dose-response reporter activity screening. 13 hits were identified from the ReFRAME library (Fig. 5a,b). The secondary dose-response screening of the top 3 hits is shown in Fig. 5c. 3 hits were identified from the ChemDiv Covalent Inhibitors library (Fig. 6a) and their dose-response activities are shown in Fig. 6b. We performed a structure activity relationship study by supplier inventory of 47 additional covalent inhibitor compounds based on the scaffolds of the top 3 hits which revealed more potent inhibitors including V011-8130 (ChoRE-LUC IC_50_ = 1.1 µM). 142 hits were identified from the 3D Pharmacophore Diversity library. Of those, 9 shared the common SD60 scaffold and 8 shared the common SC41 scaffold (Fig. 7a). Secondary dose-response screening of these hits yielded 9 confirmed hits as shown in Fig. 7b.

**Table 4:** Screening statistics for the libraries used in the primary screen.

Library	Library size	z-score cutoff	Average Z’-factor	Hits	Hit rate
ReFRAME	13122	−5.0	0.56	13	0.09%
ChemDiv 3D Pharmacophore Diversity	34658	−6.0	0.51	142	0.41%
ChemDiv Covalent Inhibitor	3574	−1.5	0.55	3	0.08%
Enzo	1638	−1.5	0.63	7	0.73%
In-house Bioactives	171	−1.5	0.60	5	2.92%

### Cytotoxicity

Hit compounds were further evaluated in dose-response for potential cytotoxicity to determine if decreased luciferase levels were not derived from the induction of cell death. Evaluation of cellular viability was measured using a CellTiterGlo assay. The top 3 most potent hits from the ReFRAME library had significant cytotoxicity as shown in Fig. 5d. For the covalent inhibitor compounds, only V029-2992 (ChoRE-LUC IC_50_ = 2.9 µM) had good potency without significant cytotoxicity (Fig. 6c). Out of the 9 hits in the 3D Pharmacophore Diversity library, 7 were not cytotoxic, with SD60-0149 and SC41-0178 being the most potent hits from their respective scaffolds (Fig. 7d).

## Data Availability

A collection of Java scripts for the analysis of our datasets have been made available on figshare with no usage restrictions^29^.
